# Stability of polymeric cationic niosomes and their plasmid DNA-based complexes as gene delivery carriers

**DOI:** 10.1080/10717544.2023.2219420

**Published:** 2023-06-15

**Authors:** Mohamed Mashal, Noha Attia, Santiago Grijalvo, Ramón Eritja, Gustavo Puras, José Luis Pedraz

**Affiliations:** a NanoBioCel Group, Laboratory of Pharmaceutics, School of Pharmacy, University of the Basque Country (UPV/EHU), Vitoria-Gasteiz, Spain; b Histology and Cell Biology Department. Faculty of Medicine, University of Alexandria, Alexandria, Egypt; c Networking Research Centre of Bioengineering, Biomaterials and Nanomedicine (CIBER-BBN), Vitoria-Gasteiz, Spain; d Institute of Advanced Chemistry of Catalonia (IQAC-CSIC), Barcelona, Spain

**Keywords:** Stability, non-viral vectors, gene delivery, cationic niosome, nioplexes

## Abstract

This study aims to explore the stability of lipo-polymeric niosomes/niosome-based pCMS-EGFP complexes under different storage temperatures (25 °C, 4 °C, and −20 °C). To date, the question of nucleic acid-complex stability is one of the most vital issues in gene delivery applications. The need for stable vaccines during the COVID-19 pandemic has merely highlighted it. In the case of niosomes as gene carriers, the scientific literature still lacks comprehensive stability studies. In this study, the physicochemical features of niosomes/nioplexes in terms of size, surface charge, and polydispersity index (PDI), along with transfection efficiency, and cytotoxicity in NT2 cells were evaluated for 8 weeks. Compared to day 0, the physicochemical features of the niosomes stored at 25 °C and −20 °C changed dramatically in terms of size, zeta potential, and PDI, while remaining in reasonable values when stored at 4 °C. However, niosomes and nioplexes stored at 4 °C and −20 °C showed nearly stable transfection efficiency values, yet an obvious decrease at 25 °C. This article provides a proof of concept into the stability of polymeric cationic niosomes and their nioplexes as promising gene delivery vehicles. Moreover, it highlights the practical possibility of storing nioplexes at 4 °C for up to 2 months, as an alternative to niosomes, for gene delivery purposes.

## Graphical Abstract

**Figure:**
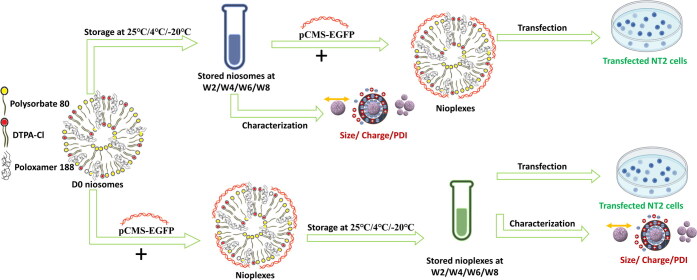

## 1. Introduction

An efficient and stable gene delivery vehicle is essential for next-generation cell-based therapy. The polyanionic nucleic acid tends to form compact structures with cationic transfection agents and hence, boosts their stability. During long-term storage of nonviral vectors, the chemical stability of their components along with colloidal physical stability are big challenges *in vitro* and *in vivo* due to the reduced bioactivity over time. Viral and non-viral carriers are the key players in gene therapy to date. In addition to their safety concerns (Attia & Mashal, 2020), the size of genetic material carried by viral vectors is limited to certain small kbs which may hamper their clinical application to deliver genes with large sizes. On the other side, non-viral vectors have a theoretically unlimited carrying capacity of the genetic material (Grijalvo et al., 2019). Nevertheless, the stability issue is still one of the main concerns. After the beginning of the COVID-19 pandemic in 2019, non-viral nanocarriers for nucleic acid-based vaccines gained a special attention. However, the stability of their formulations has emerged as an urgent topic (Shin et al., 2020). For example, the BNT162b2 mRNA COVID-19 vaccine by Pfizer and BioNTech initially has an important logistic concern as it had to be stored at −70 °C. On the other hand, Moderna’s mRNA-1273 vaccine can be stored at room temperature for up to 12 hours, in a household fridge for 30 days, and at −20 °C for up to six months (Craig et al., 2020). Recently, cationic niosomes and pegylated cationic niosomes became effective tools to condense and/or encapsulate aptamers, antisense oligonucleotides (ASOs), small interference RNAs (siRNAs), and plasmids (pDNA) (Grijalvo et al., 2019; Hemati et al., 2019). Many methods are being used to boost the stability of niosomes such as freeze-thawing, freeze-drying, and pro-niosomal dry powder (Khatoon et al., 2017). Generally, and when compared to liposomes, niosomes can overcome various difficulties related to scalability, physical stability, sterility, cost, and availability (Chen et al., 2019). Niosomes are composed of chemically stable nonionic surfactants that are less susceptible to hydrolysis or oxidation (Gupta et al., 2011; Ghanbarzadeh et al., 2015) and are able to improve the stability at different environmental conditions when compared to the phospholipid bilayer of liposomes (Perrie et al., 2007). It is reported in the literature that nonionic amphiphiles, specifically polymeric ones, enhance gene delivery by different mechanisms, and may affect the surface properties of nucleic acids (Chang et al., 2007). Additionally, the PEG chain in poloxamer 188 and polysorbate 60 tends to reduce nonspecific protein adsorption. Moreover, it improves the colloidal stability and prolongs the storage period of the formulation (Kim et al., 2022).

Nioplexes are spontaneously assembled structures composed of cationic niosomes and genetic materials. Their role as a gene delivery tool has increased over time (Ojeda et al., 2016; Grijalvo et al., 2019; Vado et al., 2021). Generally, niosomes are more stable than their liposome counterparts. The ability of niosomes to encapsulate both hydrophilic and lipophilic molecules (Mahale et al., 2012) acquired the utmost importance in gene therapy. However, the fact that niosomes are formulated in an aqueous medium raises many stability issues, mainly related to the stability of niosome structure itself (as a nano-vesicle), in order to ensure optimal consistency and reproducibility of experiments.

Although cryopreservation at −80 °C is used for the storage of liposomes (Sydykov et al., 2018), it might not be appropriate for DNA-liposome-based complexes where the addition of cryoprotectants, such as DMSO or sugar derivatives may eventually affect the transfection efficacy and cell viability. In addition, cryopreservation has damaging effects at the molecular levels, especially on the DNA molecules (Lin & Tsai, 2012). Therefore, the use of elevated subzero temperature is not only important to study the stability of DNA complexes, but also to unveil different changes in the vesicular structure related to plasmid delivery efficiency and its cytotoxicity.

The fairly water-soluble hydrochloride salt of the cationic lipid 2,3-di(tetradecyloxy)propan-1-amine (DTPA-Cl) has been used effectively for gene delivery purposes in the eye and brain (Attia et al., 2018; Mashal et al., 2019). Both poloxamer 188 (P) and polysorbate 80 (P80) nonionic surfactants along with DTPA-Cl cationic lipid (D) were used in this study to elaborate the DPP80 cationic niosomes that were able to transfect NT2 cells *in vitro* and glial cells in rat cerebral cortices, *in vivo* (Attia et al., 2018, 2019). On the other hand, DPP80 niosomes were able to transfect ARPE-19 cells *in vitro* and different rat retinal layers, *in vivo* (Mashal et al., 2019). Interestingly, NTera2/D1 teratocarcinoma-derived (NT2) cell line offers an effective strategy to deliver exogenous biological molecules into the central nervous system (CNS). Additionally, NT2 cells have the ability to differentiate into neuron-like cells (or NT2-N) that can be engrafted in the CNS of rodents and humans. Moreover, glioma tropism allows the NT2 cells to be exploited as cellular vehicles and can be genetically employed to express selected therapeutic genes (Trojanowski et al., 1993; Agirre et al., 2015; Attia et al., 2019).

Considering such promising outcomes, we tried to investigate in the present study the impact of different storage temperatures (25 °C/4 °C/-20 °C) on the stability of DPP80 niosomes and EGFP-DPP80 nioplexes. In addition, we explored the gene delivery efficiency of EGFP-DPP80 nioplexes prepared from stored DPP80 niosomes. Niosomes and nioplexes were physiochemically assayed at different storage temperatures. *In vitro* experiments were performed to evaluate the behavior of the vehicle regarding gene expression efficiency and cell viability in NT2 cells. The current study -to the best of our knowledge- is the first one to investigate the possibility of storing nioplexes (niosomes complexed with DNA) and determining the effect of storage temperature and time on their physicochemical feature and biological behavior.

## 2. Material and methods

### 2.1. Synthesis of DPP80 niosomes and nioplexes

**Figure 1.**
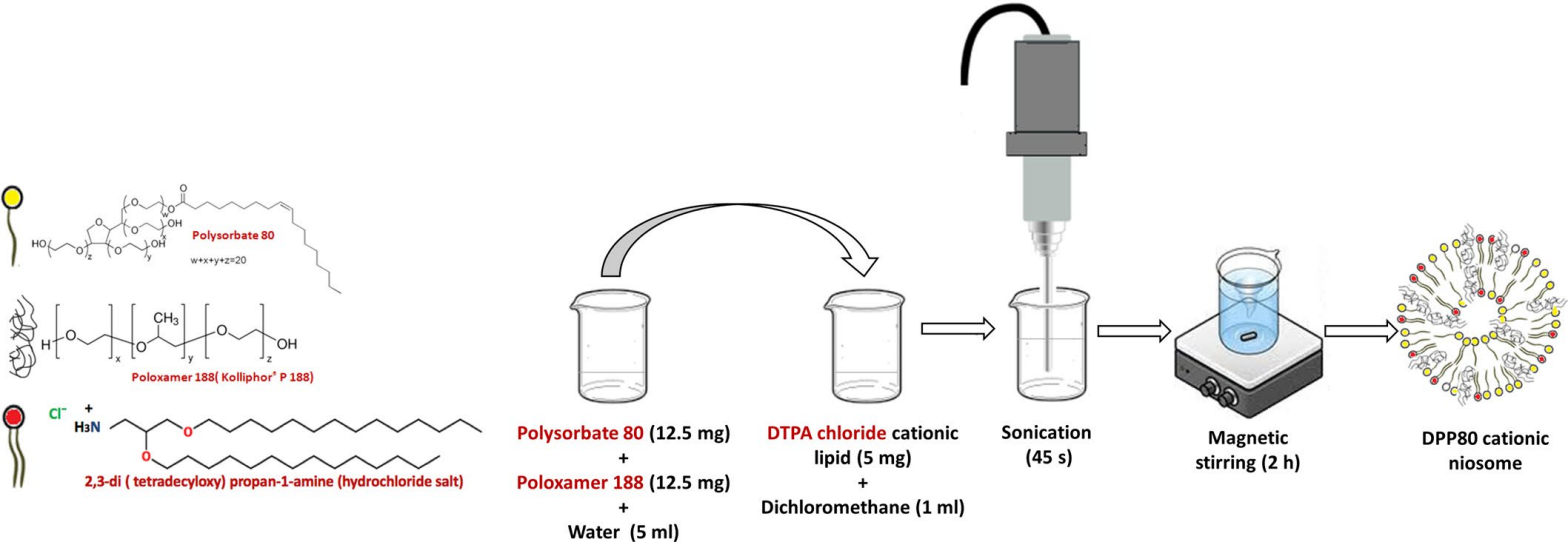
The chemical structure of DPP80 cationic niosomes [Polysorbate80 (P80), Poloxamer188 (P) and cationic lipid (DTPA-Cl) (D)] and a diagrammatic illustration for the method of niosome elaboration.

The polymeric cationic niosomes composed of the cationic lipid 2,3-di (tetradecyloxy) propan-1-amine (hydrochloride salt) (DTPA-Cl), poloxamer 188 and polysorbate 80 (at a molar ratios of 1.9: 0.3: 1.9, respectively) were prepared by the reverse phase evaporation technique (Ojeda et al., 2016). The hydrochloride salt of the cationic lipid (DTPA-Cl) was synthesized as described previously with slight modifications (Ojeda et al., 2016). Then, the organic phase of the oil-in-water nanoemulsion was prepared by dissolving 5 mg of DTPA-Cl in 1 mL of dichloromethane. Whereas the water phase consisted of 12.5 mg of P (Sigma-Aldrich, Madrid, Spain) and 12.5 mg of P80 (Sigma-Aldrich, Madrid, Spain) in 5 ml milliQ water. The nano-emulsions were obtained by sonication of the mixture for 30 s at 45 W (Branson Sonifier 250®, Danbury, USA). For 2 h under magnetic stirring, the organic solvent (dichloromethane) was removed leaving the cationic niosomes in an aqueous medium. The components and chemical structures of the cationic niosomes, and the elaboration process are represented in Figure 1.

The propagation of pCMS-EGFP reporter plasmid (5,541 bp; PlasmidFactory, Bielefeld, Germany) was reported in our previous work (Mashal et al., 2017). To get different cationic lipid/DNA ratios (w/w), different volumes of the plasmid stock solution (0.5 mg/ml) were added to various volumes of the cationic niosome suspension under a gentle vortex. The electrostatic interactions of nioplexes were enhanced by incubation for 30 min at room temperature.

The nioplexes at the cationic lipid/DNA mass ratio with best transfection results were prepared and stored to be used for the subsequent experiments. Briefly, an appropriate volume of a pCMS-EGFP plasmid stock solution was added under gentle vertexing to different volumes of the cationic niosome suspension to get different cationic lipid/DNA mass ratios (w/w). Usually, the volumes of plasmid and cationic lipids were prepared to equal volumes by the addition of serum-free Opti-MEM (Gibco^®^, Life Technologies S.A., Madrid, Spain). Then, the plasmid solution was added to the niosome solution. Their physicochemical features and transfection efficiency values in NT2 cells were elicited.

### 2.2. Cell culture protocol

Human teratocarcinoma NTera2/D1(NT2) cells (ATCC^®^-CRL,1973) were cultivated in complete medium, Dulbecco’s Modified Eagle’s Medium (DMEM) (Gibco^®^, Life Technologies S.A., Madrid, Spain), enriched with 10% fetal bovine serum (FBS) and antibiotics (100 U/ml penicillin and 100 μg/ml streptomycin (Gibco^®^, Life Technologies S.A., Madrid, Spain). Before transfection, NT2 cells were seeded in 24-well plates at an initial density of 8 × 10^4^ cells/well and allowed to grow to 70–80% confluence.

### 2.3. In vitro transfection assays

To assess the transfection activity in NT2 cells, the complete culture medium was replaced with serum-free Opti-MEM, and cells were then exposed to nioplexes (1.25 μg of pCMS-EGFP/well).

After 4 h of incubation, the transfection medium was interchanged with complete culture medium where cells were allowed to grow for 24 h before they are analyzed by fluorescence microscopy (EclipseTE2000-S; Nikon Instruments, Melville, NY, USA) and flow cytometry. The overlay images were created by the NIH ImageJ software.

The positive control **L2K** (Lipofectamine^®^ 2000, Gibco^®^, Life Technologies S.A., Madrid, Spain) was prepared following the manufacturer’s protocol. The detailed protocol was previously explained (Puras et al., 2015). NT2 cells were washed twice with PBS before being detached using 200 μl of 0.05% trypsin/EDTA. Subsequently, cells were centrifuged, and the supernatant was thrown away. The pellet was resuspended in PBS, diluted in FACSFlow liquid, and loaded directly into the flow cytometer (FACSCalibur, BD, San Jose, USA), to determine transfection efficiency (the percentage of transfected cells), cell viability (the percentage of live cells that are not stained with ethidium bromide) and mean fluorescence intensity (MFI). A preliminary experiment was initially conducted to determine the N/P mass ratio with the best transfection results that will be adopted in the subsequent stability studies.

### 2.4. Stability protocol

Niosomes and their EGFP-nioplexes at a 6/1 N/P mass ratio were stored for 8 weeks at either 25 °C, 4 °C, or −20 °C to be ready for use on time. Every other week, the niosomes were thawed and complexed to pCMS-EGFP plasmid to obtain nioplexes at a 6/1 N/P mass ratio that were subsequently used to transfect NT2 cells. On the other side, the thawed 6/1 N/P mass ratio nioplexes were used as such for the transfection and cytotoxicity assays.

### 2.5. Characterization of niosomes/nioplexes

The analysis of particle size distribution in the nano range (hydrodynamic diameter) in addition to the polydispersity index (PDI) was done by dynamic light scattering. Whereas the particles’ superficial charge (ZP) in millivolts (mV) was determined by laser doppler velocimetry (LDV) by means of the Zetasizer Nano Zs (Malvern Instrument, UK). The samples were diluted in 0.1 mM NaCl, and the reported readings were obtained by cumulative analysis. All measurements were carried out in triplicates. In addition, the morphology of nioplexes at 6/1 N/P mass ratio was assessed via the cryo-TEM using the methodology described previously (Mashal et al., 2019).

### 2.6. Statistical analysis

Statistical analysis was completed with the InStat programme (GraphPad Software, San Diego, CA, USA). The normal distribution of samples was assessed by the Kolmogorov-Smirnov test and the homogeneity of the variance by the Levene test. Differences between groups at significance levels of 95% were calculated by the ANOVA and the Student’s t-test. In all cases, *P* values < 0.05 were regarded as significant. The quantitative data were presented as mean and standard deviation unless stated otherwise.

## 3. Results

### 3.1. Physicochemical features and transfection efficiency of niosomes and nioplexes

**Figure 2.**
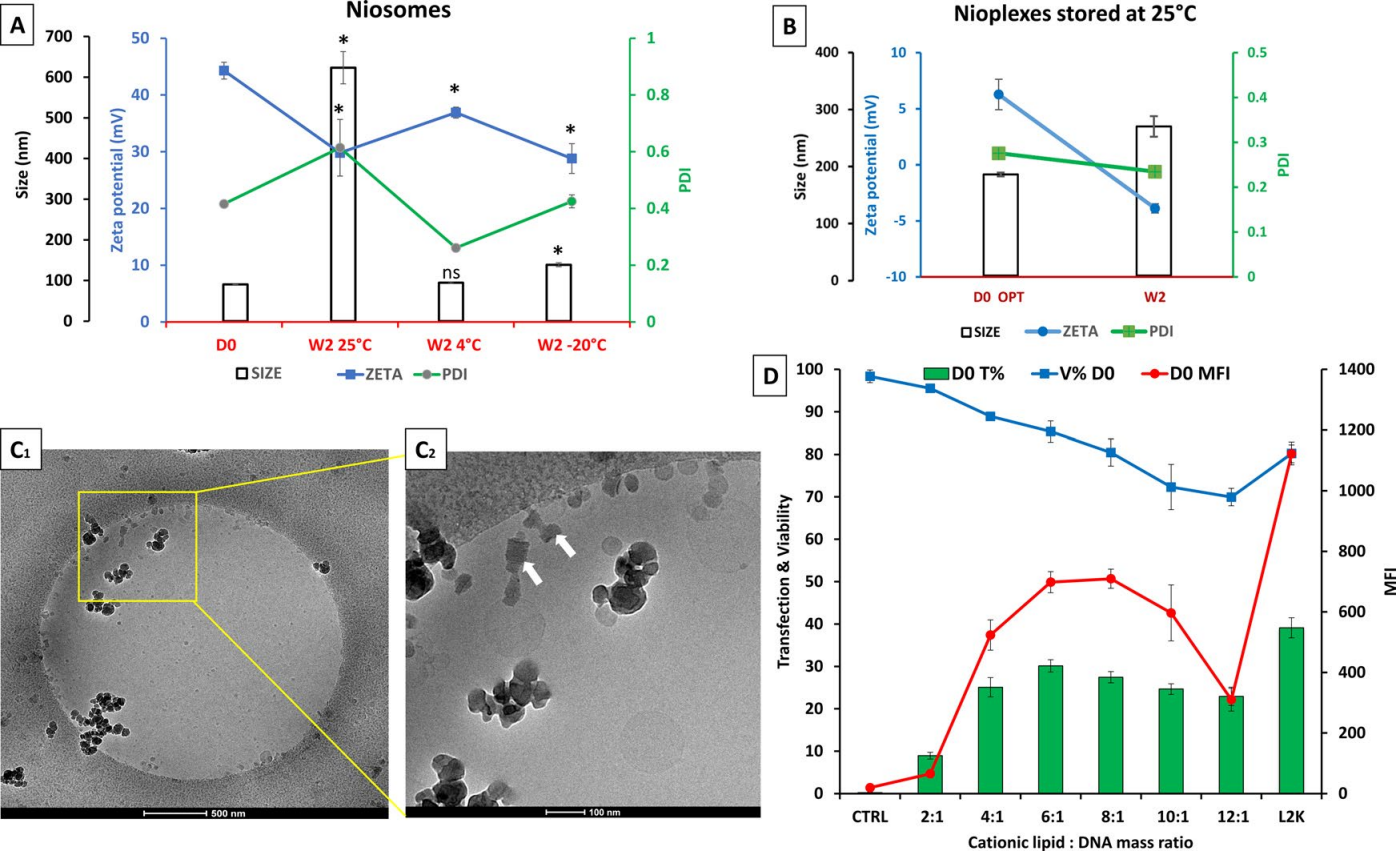
A preliminary biophysical study of DPP80 niosomes and DPP80-EGFP nioplexes. (A) the Physical features of niosomes in terms of size (nm) (bars), PDI (green line), and zeta potential (mV) (blue line) after 2 weeks of storage at 25 °C, 4 °C and -20 °C compared to day zero (D0) values. The values exemplify the mean ± SD (*n* = 3); ‘*’ indicates a significant difference compared to D0 (*P* < 0.05); ‘ns’ indicated insignificant difference. (B) The physical features of nioplexes (at a 6/1 mass ratio) in terms of size (nm) (bars), PDI (green line), and zeta potential (mV) (blue line) after 2 weeks of storage at 25 °C compared to day zero values. (C) Cryo-TEM-based morphological assessment of nioplexes at a 6/1 mass ratio. (D) Transfection of NT2 cells with nioplexes at different cationic lipid/DNA mass ratios, 24 h post-transfection. Transfection efficiency is represented as the percentage of EGFP-expressing cells (green bars), cell viability (blue line) and mean fluorescence intensity (MFI) (red line). L2K is lipofectamine^®^ 2000 (as a positive control).

**Figure 3.**
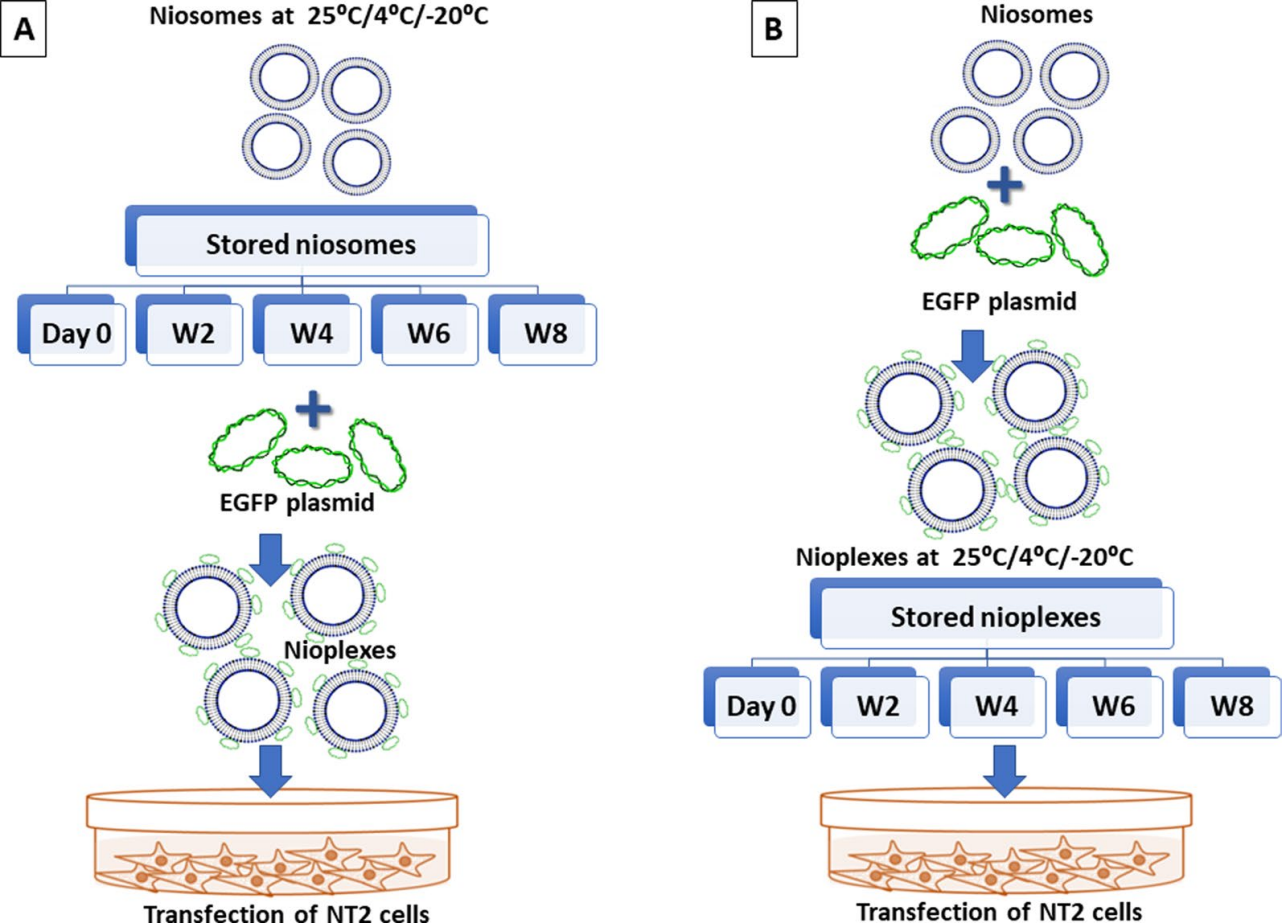
A Schematic representation of the experimental study design. (A) Represents the storage of niosomes at 25 °C, 4 °C and -20 °C up to 8 weeks. The thawed niosomes are complexed with pCMS-EGFP at a 6/1 N/P mass ratio to form nioplexes to transfect NT2 cells. (B) The freshly prepared niosomes are immediately complexed with pCMS-EGFP at a 6/1 N/P mass ratio that are stored later at 25 °C, 4 °C and -20 °C for up to 8 weeks. The thawed nioplexes are used to transfect NT2 cells.

Figure 2 exhibits the results of a preliminary biophysical study of niosomes and nioplexes. Figure 2(A) shows the physical features of DPP80 cationic niosomes in terms of size (nm) (bars), polydispersity index (PDI) (green line), and zeta potential (mV) (blue line) after 2 weeks of storage at 25 °C, 4 °C and −20 °C compared to day zero values. It was obvious that only after 2 weeks of storage at 25 °C, the size of niosomes (Figure 2(A)), and nioplexes (Figure 2(B)) showed a drastic increase, while the zeta potential values were markedly decreased. Therefore, we did not continue with stability experiments for niosomes/nioplexes at the storage temperature of 25 °C till week 8. As revealed in Figure 2(C), the morphology of nioplexes at 6/1 mass ratio by cryo-TEM, depicted multi-lamellar complexes (white arrows) which appear in distinct spherical, and/or aggregated lamellar patterns. Regarding the transfection efficiency (in terms of the percentage of GFP-expressing cells and their MFI) the nioplexes at a 6/1 N/P mass ratio depicted the best transfection efficiency compared to the other N/P mass ratios tested (Figure 2(D)). In addition, this particular N/P mass ratio did not depict alarming cytotoxicity. Therefore, in the following transfection experiments, the N/P mass ratio of 6/1 was adopted, and then the stability protocol was followed as depicted in Figure 3. At either 25 °C, 4 °C, or −20 °C, both niosomes and the corresponding nioplexes at a 6/1 N/P mass ratio were stored for 8 weeks. Hence, physicochemical characterization along with the biological performance in NT2 cells were assessed every 2 weeks.

### 3.2. Physicochemical characterization of niosomes stored at 25 °C, 4 °C and -20 °C

**Figure 4.**
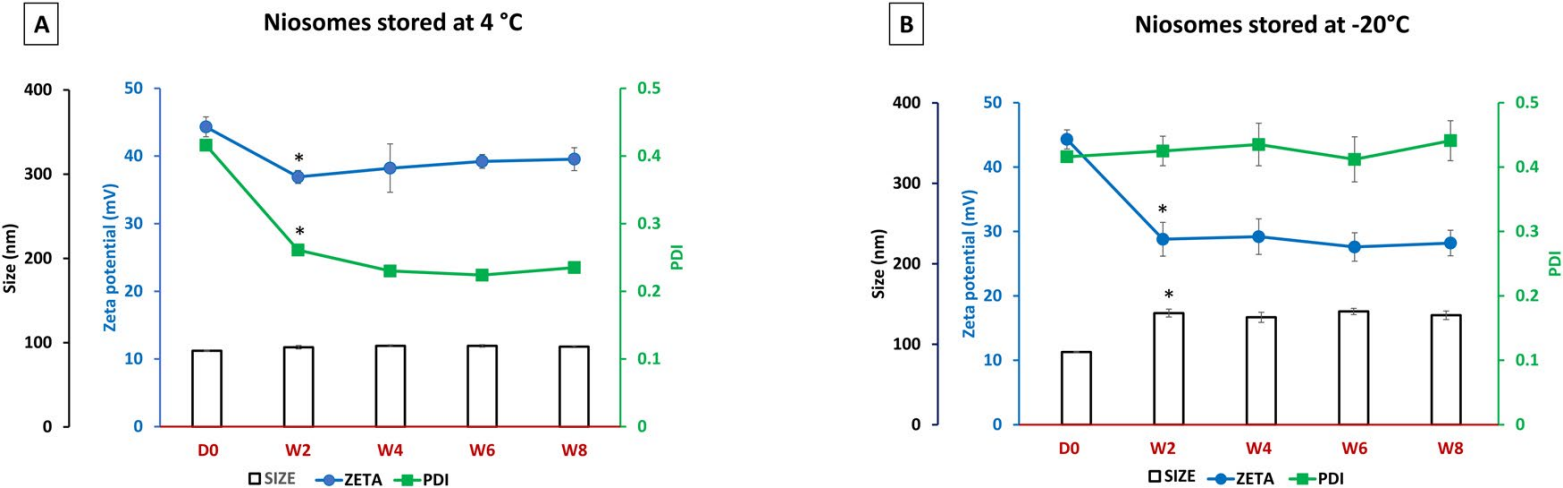
Physicochemical characterization of niosomes stored at 4 °C (A) and at -20 °C (B) over time (day zero up to week 8) in terms particle size (bars), zeta potential (blue line) and PDI (green line). Each data point represents the mean ± SD (*n* = 3); ‘*’ indicates a significant difference compared to D0 (*P* < 0.05).

Figure 4(A) illustrated the change in size (bars), zeta potential (blue line), and PDI (green line) of niosomes stored at 4 °C and assessed biweekly for 8 weeks. Compared to day 0 values, there was a significant decrease in the zeta potential and PDI values of the stored niosomes after 2 weeks of storage (*P* < 0.05), albeit a slight increase in their size values (*P* > 0.05). However, the change in the zeta potential and PDI parameters for the stored niosomes displayed a near-plateau profile till the end of the study (week 8). On the other side, Figure 4(B) depicted the change in the same parameters for niosomes stored at −20 °C. The size increased after 2 weeks (*P* < 0.05) and then slightly fluctuated between about 133 nm to 141 nm during the storage period (*P* > 0.05). In contrast, zeta potential values decreased dramatically after 2 weeks (*P* < 0.05) before displaying an obvious stable profile (ranging between 27 to 29 mV) until week 8 (*P* > 0.05). Interestingly, the PDI values were stable throughout the experiment, around the level of 0.4.

### 3.3. Biological performance of niosome stored at 25 °C, 4 °C and -20 °C

**Figure 5.**
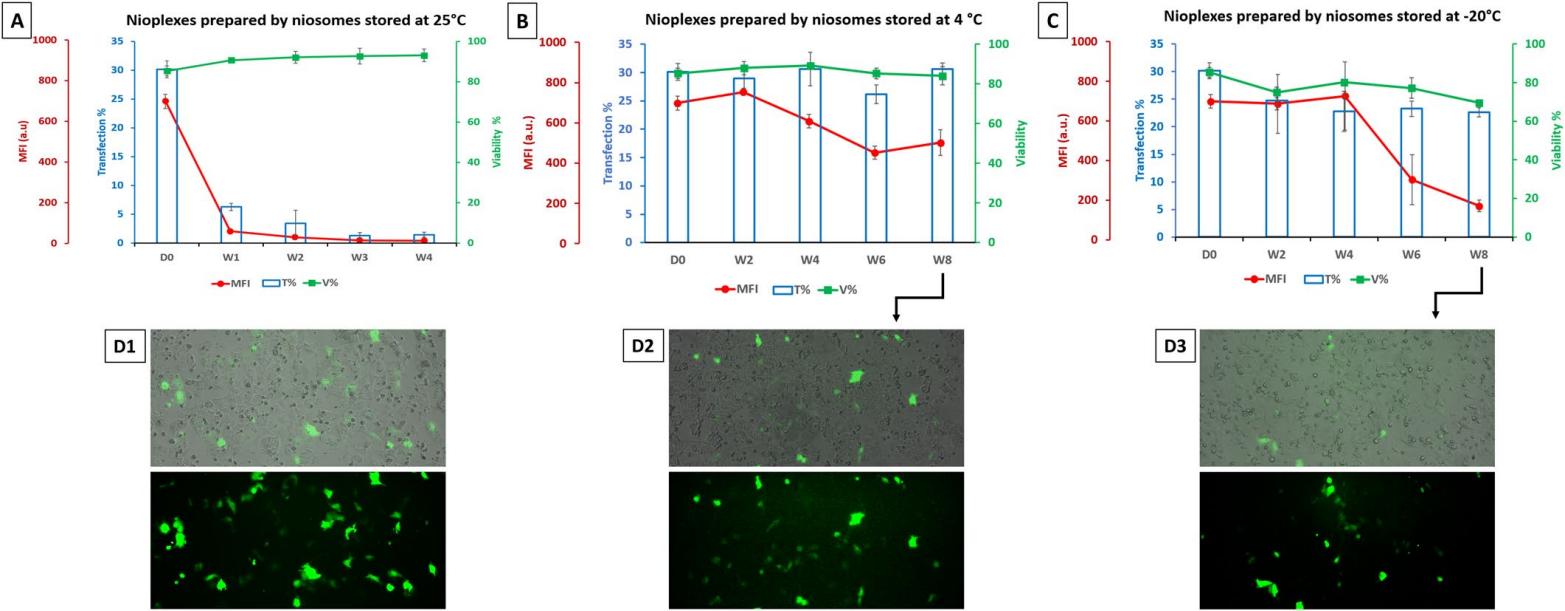
(A) Biological performance of 6/1 nioplexes prepared by niosomes stored at 25 °C, (B) at 4 °C and (C) at -20 °C, in terms of transfection efficiency (bar graphs), cell viability (green line graphs) and MFI (red line graph). Each data point represents the mean ± SD (*n* = 3). (D) Overlay of fluorescence and phase-contrast micrographs of NT2 cells transfected with nioplexes prepared by day zero niosomes (D1), and nioplexes prepared by niosomes stored for 8 weeks at 4 °C and -20 °C (D2 and D3, respectively). Original magnification x100.

Figure 5 shows the biological performance in terms of transfection efficiency, viability, and MFI in NT2 cells of nioplexes prepared by niosomes stored at 25 °C, 4 °C and −20 °C. All transfection experiments on NT2 cells used the N/P mass ratio of 6/1.

Figure 5(A) shows the biological performance of nioplexes prepared by niosomes stored at 25 °C for 4 weeks compared to day zero (D0). The results depicted a marked decrease in transfection efficiency (in terms of the percentage of transfected cells and MFI) over time. Contrary to the transfection efficiency, all the viability values were less affected by time. Due to the unsatisfactory results at 25 °C, the transfection experiments were stopped at this point (week 4). Interestingly, the nioplexes prepared by the niosomes stored at 4 °C (Figure 5(B)) showed viability values close to those of day zero (84-89%). Moreover, the percentage of transfected cells fluctuated within a narrow range (26-31%), while the MFI values started to decrease to relatively lower values from week 4. Finally, Figure 5(C) depicted the transfection efficiency, MFI, and viability of NT2 cells transfected with nioplexes prepared by the −20 °C stored niosomes. Although the transfection efficiency decreased markedly after 2 weeks compared to day zero values (*p* < 0.05), the values fluctuated within a small range in the following weeks (about 23 to 25%). In addition, the NT2 cell viability values decreased from 70 to 80% during the study duration. Regarding the MFI, the values remained stable till week 4 and dropped afterwards. Representative micrographs depict NT2 cells transfected with nioplexes prepared by day zero (D0) niosomes (Figure 5(D1)), and nioplexes prepared by niosomes stored for 8 weeks at 4 °C and −20 °C (Figure 5(D2,D3), respectively).

### 3.4. Physicochemical characterization of nioplexes stored at 25 °C, at 4 °C and -20 °C

**Figure 6.**
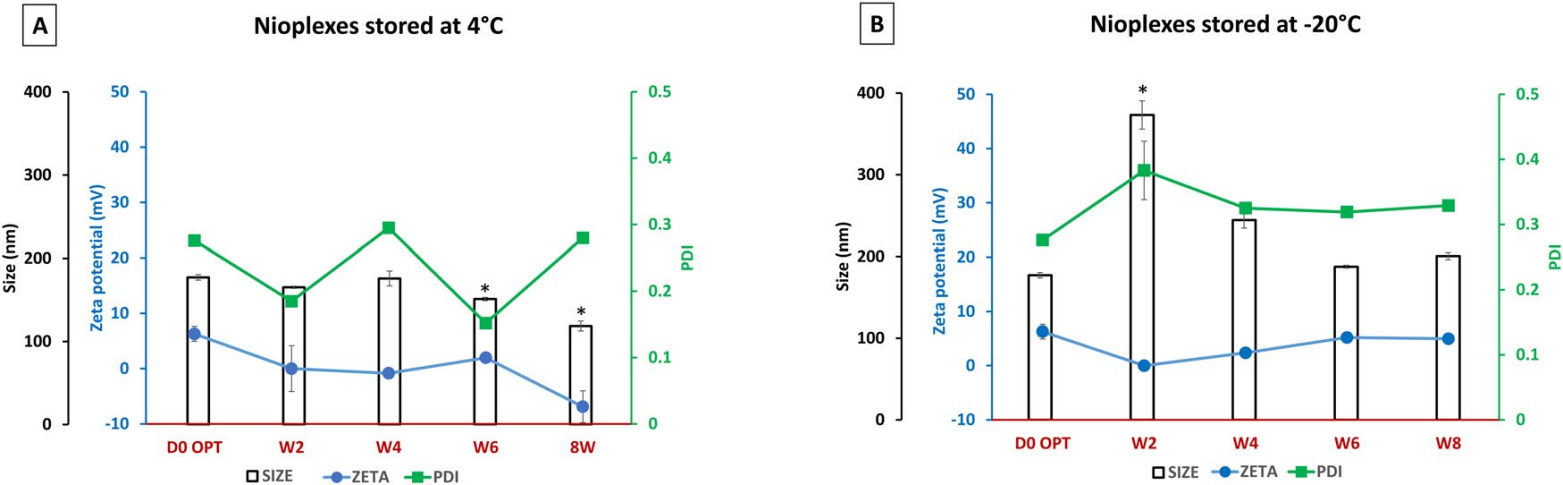
(A) Physicochemical characterization of nioplexes stored in Opti-MEM at 4 °C and (B) at -20 °C over time (day zero D0 up to week 8) in terms particle size (bars), zeta potential (blue line) and PDI (green line). Each data point represents the mean ± SD (*n* = 3); ‘*’ indicates a significant difference compared to D0 (*P* < 0.05).

The nioplexes, prepared using freshly prepared niosomes at a 6/1 N/P mass ratio in an Opti-MEM solution and stored at 25 °C, at 4 °C and −20 °C. The day zero readings were about 177 nm, 6 mV and 0.27 for size, zeta potential and PDI, respectively (Figure 6).

The physicochemical measurements of the nioplexes stored at 25 °C were discussed before in Figure 2(B). At 4 °C (Figure 6(A)), the sizes were quite similar to day zero readings except at weeks 6 and 8 which decreased to 151 and 118 nm, respectively. Regarding the zeta potential values, they fluctuated within a small range and were close to zero mV. The PDI values fluctuated within a range of 0.15 to 0.29. On the other side, when nioplexes were stored at −20 °C (Figure 6(B)), they depicted a marked increase of the tested physicochemical parameters when compared to the readings at 4 °C (Figure 6(A)). The size was about 373 nm at week 2, then decreased to about 200 nm by week 8. Likewise, the zeta potential values fluctuated within a narrow range in the positive territory (about 2-5 mV) except at week 2 (about −1.17 mV). In addition, the PDI readings were around 0.3 throughout the experiment.

### 3.5. Biological performance of nioplexes stored at 25 °C, 4 °C and -20 °C

**Figure 7.**
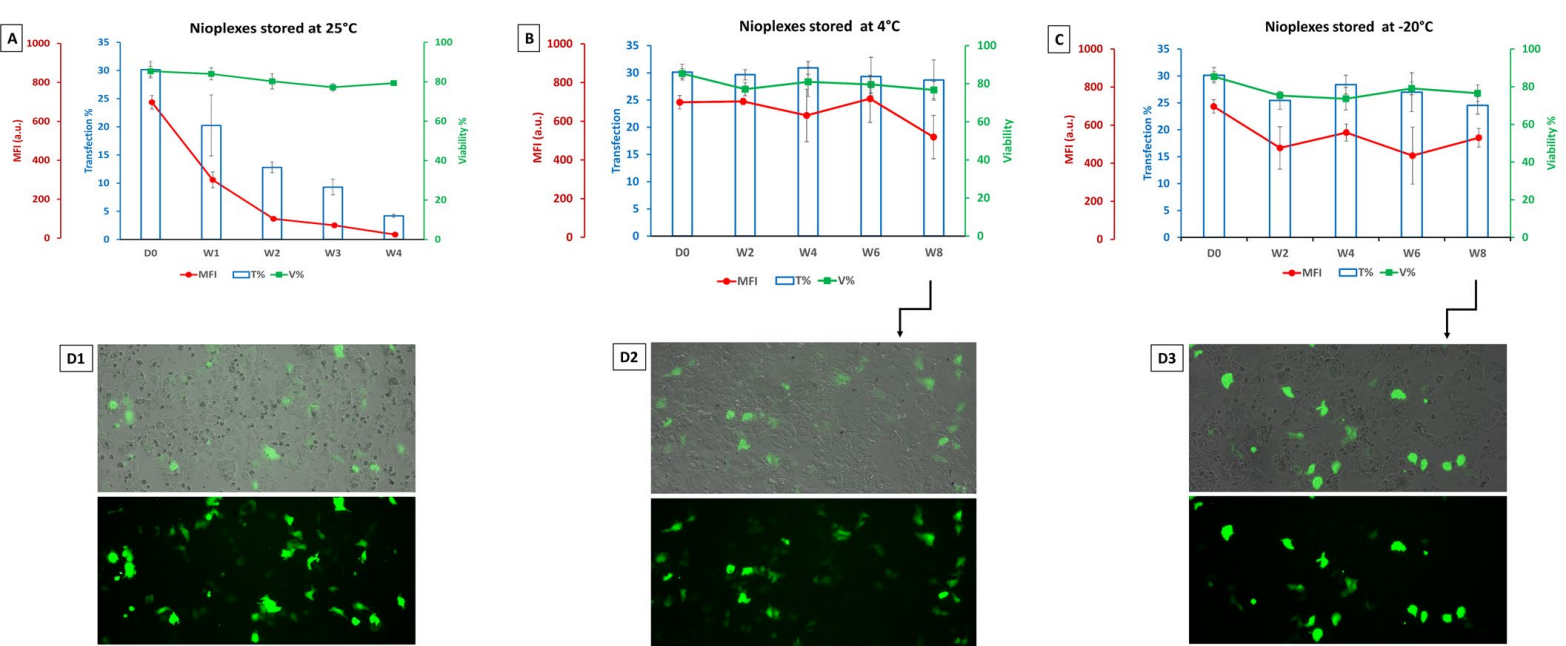
(A) Biological performance of nioplexes stored at 25 °C, (B) at 4 °C and (C) at -20 °C, in terms of transfection efficiency (bar graphs), cell viability (green line graphs) and MFI (red line graph). Each data point represents the mean ± SD (*n* = 3). (D) Overlay of fluorescence and phase-contrast micrographs of NT2 cells transfected with day zero (D0) nioplexes (D1), and nioplexes stored for 8 weeks at 4 °C and -20 °C (D2 and D3, respectively). Original magnification x100.

Figure 7 shows the biological performance of nioplexes at 6/1 mass ratio stored at 25 °C, 4 °C and −20 °C in terms of transfection efficiency, viability, and MFI in NT2 cells. The nioplexes stored at 25 °C depicted a marked decrease in transfection efficiency over time (Figure 7(A)). In addition, the viability values were slightly decreasing. Regarding the nioplexes stored at 4 °C (Figure 7(B)) and compared to day zero (D0), the transfection percentage and cell viability remained almost steady over time. Regarding the MFI, the values fluctuated within a small range until week 6 when compared to day zero, while decreased by week 8 to about 518. Figure 7(C) shows NT2 cells transfected with nioplexes stored at −20 °C, the transfection percentage was about 25-28% over the duration of the study (8 weeks) (slightly less than day zero). Likewise, the cell viability fluctuated between 74 and 79% (slightly less than day zero *p* > 0.05). Similarly, the MFI fluctuated between 480 and 560 a.u. which is significantly less than the value at day zero (∼ 698 a.u., *P* < 0.05). Representative micrographs depict NT2 cells transfected with day zero nioplexes (Figure 7(D1)), and nioplexes stored for 8 weeks at 4 °C and −20 °C (Figure 7(D2,D3), respectively).

## 4. Discussion

As demonstrated in other publications, our in-house DPP80 niosomes offer a great potential as a non-viral gene delivery vehicle to the brain and the retina (Attia et al., 2018; Mashal et al., 2019). However, in general, lipidic nano-formulations in aqueous suspensions tend to be less stable due to the presence of excess water molecules. Subsequently, this could lead to rapid hydrolytic degradation of those lipid preparations (Heurtault et al., 2003). The reverse phase evaporation method (illustrated in Figure 1) is widely used for the production of large unilamellar vesicles (LUVs) (Homaei, 2016). Moreover, the high hydrophilic-lipophilic balance (HLB) value of DPP80 niosomes (∼ 16.8), as calculated by the Davies’ equation (Davies, 1957), is deemed the reason behind large vesicle formation (Shaker et al., 2017). On the other hand, the sonication time is crucial to obtain small nano-sized vesicles (< 100 nm). As a result, the free energy of the system increases, which can lead to agglomeration due to thermodynamic instability of the system. Hereafter, the incorporation of nonionic surfactants (e.g. polysorbate 80, poloxamer 188, etc.) enhances electrostatic repulsion between nano-vesicles, thus minimizes their agglomeration. Moreover, the steric hindrance introduced by poloxamer 188 is believed to enhance the vesicular stability, hence grants a long-term stability for niosome and nioplexes. Therefore, electrostatic stability is believed to be synergistically achieved by combining the polysorbate 80 along with poloxamer 188 in the synthesis of niosomes (Attia et al., 2018). Moreover, the *in vivo* studies conducted on nucleic acid vaccines reveal the importance of polymeric materials on the enhanced stability, cellular uptake, endolysosomal escape, gene expression, and biosafety of the genetic material (Chen et al., 2022).

In the current study, the cationic lipid/DNA mass ratio of 6/1 was found to be the one with the best transfection efficiency in NT2 cells. Therefore, the same mass ratio was adopted in the following transfection experiments. One of the most critical parameters that affects transfection efficiency is the selection of transfection buffer. In this regard, Opti-MEM was found to improve cell viability and transfection when compared with other buffers, such as PBS (Hyder et al., 2020). As a result of being a reduced serum medium ideal for cationic lipid transfections, we chose Opti-MEM as a transfection medium for NT2 cells (Attia et al., 2018, 2019). Moreover, we opted to use it in the preparation and storage nioplexes. However, comparing different buffered media to store the nanocomplexes merits further investigation. Buffered solutions are known to change the conformational behavior, neutralize DNA charge, lower the electrophoretic mobility, and enhance compaction of DNA chain (Savelyev & Papoian, 2006). Using a buffer seems to be crucial factor in the effectiveness and physical properties outcomes of the nioplexes formulation. The ionic strength and counter ions of Opti-MEM plays competitive and cooperative roles in the electrostatic binding process, and sometimes in counter ion complexation (Wang et al., 2018). Unlike buffered solutions with high ionic strength (PBS) which can weaken the complex condensation process, Opti-MEM with its lower ionic strength could enhance cationic lipid to DNA binding (Pozharski & MacDonald, 2002). Therefore, DNA and cationic lipid complexes could be prepared at low ionic strength (Felgner, 1991). In general, the alteration of the zeta potential of nioplexes to small values when compared to niosomes, can be considered an indication of successful electrostatic binding between the nucleic acids and the niosomes (Al Qtaish et al., 2020).

The niosomes stored at 4 °C depicted a nearly stable size until week 8 (Figure 4(A)). The marked decrease in PDI value during the first two weeks could be due to the aggregation of extremely small niosome population into mean-size niosome (Sezgin-Bayindir & Yuksel, 2012). The same stability profile was detected with niosomes stored at −20 °C, albeit with much lower zeta potential values (Figure 4(B)). Most likely, during freeze-thawing, many changes at the nano level tend to occur which result in an alteration of the zeta potential. Besides, both the crystallization of the external phase, the solidification, and the change of the lipid phase could have a tremendous effect on these physicochemical properties (Choi et al., 2004). It is important to point out that the nano-complex formation with the addition of DNA would totally change the physicochemical profile of the new nanoparticles as a result of strong electrostatic interaction between negative DNA macromolecules and positive charge on the cationic niosomes. Nonetheless, the basic definition of stability of an emulsion is the ability of the system to resist changes in its physicochemical properties over time. However, in gene delivery, those changes might have a direct positive impact on the effectiveness of the vector to transfect cells (Grimaldi et al., 2016). The random re-arrangement of the complex after the addition of DNA to the cationic vesicles is affected by changes in those vesicles over time. Therefore, the mechanistic investigation of the disintegration of each component of the cationic vesicles may be a key determinant factor of their efficacy over time to denote whether the changes in the physicochemical properties become in favor of its transfection efficacy or not. The actual mechanism of DNA’s electrostatic interaction remains poorly understood. However, this process is thought to be driven mainly by the existence of counterions on the surface of DNA (Besteman et al., 2007).

In the current work, the size readings of niosomes and nioplexes stored at 4 °C were in general more stable than their counterparts stored at −20 °C (Figures 4 and 6). On the other side, the readings of zeta potential were generally decreasing over time which could be due to many factors such as van der Waals forces, electrostatic interaction, physical/chemical degradation, temperature, pH, salt concentration, and the presence of biological fluids in the storage medium (Freitas & Müller, 1998). It is worth noting that, the zeta potential of nioplexes stored at 4 °C and −20 °C fluctuated within a narrow range close to zero (Figure 6) which highlights the favorable storage stability of nioplexes over niosomes.

Despite the pro-stability high polymeric content of the current niosome formulation, the ice crystals formed during freezing might damage the niosome vesicles, resulting in their rupture, aggregation, or expansion (Seneviratne et al., 2020). A similar change in physical characteristics was seen in liposomal vesicles that underwent freezing at −22 °C (Costa et al., 2014). Nevertheless, those liposomes showed stability at temperatures above 0 °C (Costa et al., 2014). Likewise, the niosomes and nioplexes stored at 4 °C maintained satisfactory stability in terms of cell viability and transfection efficiency (Figures 5(B) and 7(B)). The stability of stored niosomes/nioplexes provides experimental reproducibility with the advantage to retest the same patch for further comparative studies. Interestingly, in terms of their biological performance in vitro, the nioplexes stored at 4 °C demonstrated a more stable profile compared to nioplexes prepared from niosomes stored at 4 °C (Figure 5). Generally, the storage of nano-emulsion at the fridge temperature (4 °C) could hold better stability than storage at room temperature (25 °C). Compared to the storage temperature of 4 °C, both frozen nioplexes and nioplexes prepared from niosome stored at −20 °C depicted comparable, yet less stability (Figures 5 and 7). Despite the decrease in MFI after week 4, in case of stored niosome compared to stored nioplexes, this might not be an alarming finding in this setting for two main reasons; First, the amount of transfected protein expression (GFP in this case) is expected to be functioning in low amount. Second, the percentage of GFP expression to cell viability should be taken in consideration. Furthermore, the MFI and transfection percentage are two completely different parameters that should not be expected to positively correlate all the time. While the transfection percentage indicates the percentage of transfected cells, the MFI indicates the mean values of the expressed GFP (in arbitrary units) which is merely an indirect indicator for transfection, and should be taken with a pinch of salt (Breunig et al., 2008).

Although the nioplexes stored at −20 °C showed an appealing biological stability pattern (Figure 7(C)), it could not beat that seen with nioplexes stored at 4 °C (Figure 7(B)). However poloxamer 188, as a polymeric stabilizer, did not improve the stability of nano-suspensions at room temperature, it managed to enhance it under refrigerated conditions (Lestari et al., 2015). The nonionic polymer at low temperatures is able to impede particle size from undergoing the Ostwald ripening, and hence prevents nanoparticle aggregation (Lestari et al., 2015). It is noteworthy that, the changes in physical characteristics of vesicles by freezing can lead to a decrease/increase in the transfection efficiency. Sork and colleagues reported that freezing and thawing of Lipofectamine 2000^®^ could alter the membrane structure, and consequently the shape of the complex and enhance its gene delivery efficacy without compromising the viability in many cell lines (Sork et al., 2016). This might explain the different results of transfection efficiency and cell viability upon the use of frozen niosomes/nioplexes. Considering the cell viability results in this study, the only marked decrease of cell viability over time was noticed when niosomes stored at −20 °C were used (Figure 5(C)) which was concomitant with a decreased in the MFI. Unfortunately, the currently available data in the literature could not clearly explain the actual mechanism behind such increase in cytotoxicity. However, this might be due to the inherent changes occurring in the niosomes upon freezing such as fusion of vesicles or aggregate formation, leakage of component, hydrolysis and/or oxidation of lipids (Boafo et al., 2022). This again should emphasize the sturdy structure of nioplexes that can withstand subzero temperatures better than niosomes.

The results obtained suggest the absence of correlation between the change in physicochemical properties and transfection efficiency of stored niosomes and nioplexes at −20 °C. This remains a paradox as formulations without stable physicochemical feature could still retain their transfection efficiency. The overall pattern of storge stability values showed the ability of frozen nioplexes to introduce a possible option of preservation of nioplexes for longer periods of time. Although, it should remain clear that repeated cycles of freezing and thawing of niosomes should be avoided as they can induce dramatic change in their size (Sriwongsitanont & Ueno, 2010; Bartelds et al., 2018).

To conclude, as a proof of concept, this study highlights the possibility to store nioplexes, as another promising alternative to niosomes, at 4 °C for up to 2 months for gene delivery purposes. The sturdy structure of nioplexes, compared to niosomes, introduces a logic translational outcome from bench-top to bedside in the field of gene therapy. The storage of niosomes and nioplexes at 25 °C rendered the particles unstable after one week. Likewise, drastic changes in physicochemical parameters were observed when niosomes and nioplexes stored at −20 °C. As a result, freezing at this temperature did not provide a favorable option for storage. On the other hand, in the current study, niosomes and nioplexes stored at 4 °C depicted the best stability option for almost two months. It is noteworthy that the behavior of stored niosomes and nioplexes (in terms of transfection efficiency and cytotoxicity in NT2 cells) was found not to correlate with the stability of their physicochemical features. However, this point definitely merits further research.

## Data Availability

The data that support the findings of this study are available from the corresponding author, [José Luis Pedraz], upon reasonable request.
